# Effects of physical activity on eating disorder tendencies in college students: the chain-mediated effects of trait positivity and physical self-esteem

**DOI:** 10.3389/fpsyg.2025.1588744

**Published:** 2025-07-14

**Authors:** Yong Jiang, XiangZheng Meng

**Affiliations:** School of Physical Education, Liaoning Normal University, Dalian, China

**Keywords:** physical activity, eating disorder tendencies, trait positivity, physical self-esteem, the chain-mediated effects

## Abstract

**Aim:**

This study aimed to explore the influence mechanism of physical activity on eating disorder tendencies in college students and analyze the chain-mediated role of trait positivity and physical self-esteem.

**Methods:**

Using the Physical Activity Rating Scale, Eating Disorder Tendency Scale, Trait Positivity Scale, and Physical Self-Esteem Scale, 550 college students from four universities in Northeast China were surveyed.

**Results:**

(1) Physical activity was significantly negatively correlated with eating disorder tendencies in college students (*r = −*0.361*; p <* 0.01); trait positivity was significantly negatively correlated with eating disorder tendencies in college students (*r =* −0.275*; p <* 0.01); physical self-esteem was significantly negatively correlated with eating disorder tendencies in college students (*r =* −0.436*; p <* 0.01); physical activity was significantly positively correlated with trait positivity (*r =* 0.223*; p <* 0.01); physical activity was significantly positively correlated with physical self-esteem (*r =* 0.371*; p <* 0.01); trait positivity was significantly positively correlated with physical self-esteem (*r =* 0.433*; p <* 0.01). (2) Physical activity could negatively predict eating disorder tendencies in college students, with a direct effect of −0.0202. (3) In the association between physical activity and eating disorder tendencies in college students, trait positivity and physical self-esteem could exert both separate mediating effects and a chain-mediated effect, with effect values accounting for 4.02, 17.23, and 4.76% of the total effect, respectively.

**Conclusion:**

Physical activity is associated with eating disorder tendencies in college students, and this association occurs both through the mediating roles of trait positivity and physical self-esteem and through their chain-mediated effect.

## 1 Introduction

Eating disorder tendencies refer to abnormal dietary behaviors that not only pose serious threats to individuals’ physical health but may also trigger psychological problems including depression and anxiety, and even lead to self-harm and suicidal thoughts (Wei, 2025). The causes of eating disorder tendencies are complex, involving physiological, familial, psychological, and social dimensions (Weems, 2023). College students, as a special group undergoing critical life transitions, experience significant physiological, psychological, and social role changes during this period (Ning and Yu, 2024). Although possessing higher education levels and self-awareness, they simultaneously endure pressures from academics, career planning, and interpersonal relationships (Yang H. H., 2024). Consequently, the detection rate of eating disorder tendencies in college students is relatively high and shows an increasing trend. Specifically, between 2011 and 2021, the detection rate was significantly higher than during 2002–2010, highlighting the severity of this issue (Liu et al., 2022). Physical activity plays a crucial role in mitigating eating disorder tendencies by enhancing physical health, regulating the relationship between perfectionism and eating disorder tendencies, improving psychological well-being, promoting positive physical self-evaluation, and reducing abnormal eating behaviors (Zhang et al., 2018). However, some studies indicate that participation in certain specific types of exercise may exacerbate individuals’ concerns about physical appearance, thereby adversely affecting recovery from eating disorder tendencies (Wang et al., 2022). Physical self-esteem is an individual’s subjective evaluation of their body: high levels lead to positive acceptance, while low levels may trigger negative emotions. Trait positivity, a stable personality trait, reflects constant and automatic awareness of present experiences. Both trait positivity and mindfulness emphasize present-moment awareness and non-judgment for mental health benefits. The key difference is that trait positivity is an inherent personal tendency, while mindfulness relies on systematic training. Therefore, in-depth research on how physical activity reduces eating disorder tendencies in college students by enhancing physical self-esteem and trait positivity is essential for promoting physically and mentally healthy development in this population.

## 2 The chain-mediated hypothetical model of the relationship between physical activity and eating disorder tendencies in college students

### 2.1 Predictive effects of physical activity and the eating disorder tendencies in college students

Physical activity is a form of bodily movement involving planned, structured, and repetitive physical actions aimed at improving or maintaining one or more components of physical health (American College of Sports Medicine, 2018). Physical activity encompasses not only vigorous forms of exercise but also gentle movement modalities that facilitate emotional relaxation, cultivate temperament, and enhance psychological well-being (Huang et al., 2013). By promoting healthy lifestyles, Physical activity can not only prevent eating disorder tendencies but also foster appreciation for one’s body, eliminate negative fears about appearance, and strengthen individuals’ sense of value and meaning through its fitness and educational function (Raisi et al., 2023). Furthermore, habitual engagement in Physical activity helps college students integrate into sports communities, experience the pleasure derived from movement, and enhances their capacity to cope with setbacks and anxiety (Qin and Qin, 2023). As a purposeful bodily endeavor, physical activity fundamentally aims to improve or sustain physical health levels (American College of Sports Medicine, 2011). Based on this, we propose hypothesis H1: Physical activity negatively predicts eating disorder tendencies in college students.

### 2.2 Prediction of the mediating effect of trait positivity

Trait positivity refers to an individual’s ability to maintain awareness and focus on present-moment experiences (Brown and Ryan, 2003). It is influenced by both innate factors and acquired factors such as education and interventions (Duan, 2014). Trait positivity and mindfulness both focus on awareness and concentration to enhance mental health, with the former being a stable personality trait and the latter an acquired practice. Training to enhance trait positivity has been shown to alleviate physical discomfort in patients with anorexia and help individuals break free from the control of emotions and thoughts, reducing binge eating behaviors (Wang et al., 2021).

Trait positivity also improves self-control over eating behaviors, enhances food awareness, and modifies unhealthy dietary habits (Liang et al., 2022). Individuals with high levels of Trait Positivity typically exhibit stronger emotional regulation and acceptance abilities. Even after overeating, they can regulate subsequent negative emotions and reduce binge eating or purging behaviors (Hussain et al., 2021). Physical activity is recognized as being associated with individuals’ trait positivity levels, enabling participants to focus more intently on present-moment sensations during exercise and derive satisfaction from the process (Xu, 2021). Based on this, we propose hypothesis H2: Trait positivity negatively predicts eating disorder tendencies in college students, thereby playing a mediating role in the relationship between physical activity and eating disorder tendencies in college students. This process not only promotes physical health but also provides college students with a comprehensive pathway for preventing and intervening in eating disorder tendencies.

### 2.3 Prediction of the mediating effect of physical self-esteem

The other mediator selected in this study is physical self-esteem. Physical self-esteem refers to individuals’ satisfaction with their bodies, encompassing physical attractiveness, athletic skills, physical condition, and fitness (Fox and Corbin, 1989). Individuals with higher physical self-esteem typically demonstrate stronger emotion regulation abilities (Ma and Wang, 2021). Existing research indicates a significant association between physical self-esteem and eating disorder tendencies in college students: higher physical self-esteem correlates with lower eating disorder tendencies in college students, reduced body dissatisfaction, and fewer pathological eating behaviors (Li, 2022). Simultaneously, positive bodily perception and acceptance can mitigate the risk of eating disorder tendencies in college students (Wang et al., 2020). Studies confirm that physical activity serves as a crucial means to enhance physical self-esteem (Liao and Li, 2024), effectively promoting its development among students. This process strengthens self-identity, builds greater self-confidence, and enables more flexible and proactive responses to adversity (Li et al., 2019).

Physical self-esteem is essential for psychological well-being and life satisfaction, and its enhancement remains a key focus in university mental health initiatives (Sun and Zhang, 2020). As a multidimensional evaluation of one’s physical condition, attractiveness, and other factors, physical self-esteem is closely linked to bodily movement. Multiple studies have verified physical activity’s significant positive predictive effect on physical self-esteem—that is, regular physical activity promotes higher physical self-esteem levels (Zhu and Chen, 2009; Jia et al, 2018). Based on this, we propose Hypothesis H3: Physical self-esteem negatively predicts eating disorder tendencies in college students, thereby playing a mediating role in the relationship between physical activity and eating disorder tendencies in college students.

### 2.4 Prediction of the chain-mediated effect of trait positivity and physical self-esteem

**Figure 1:**
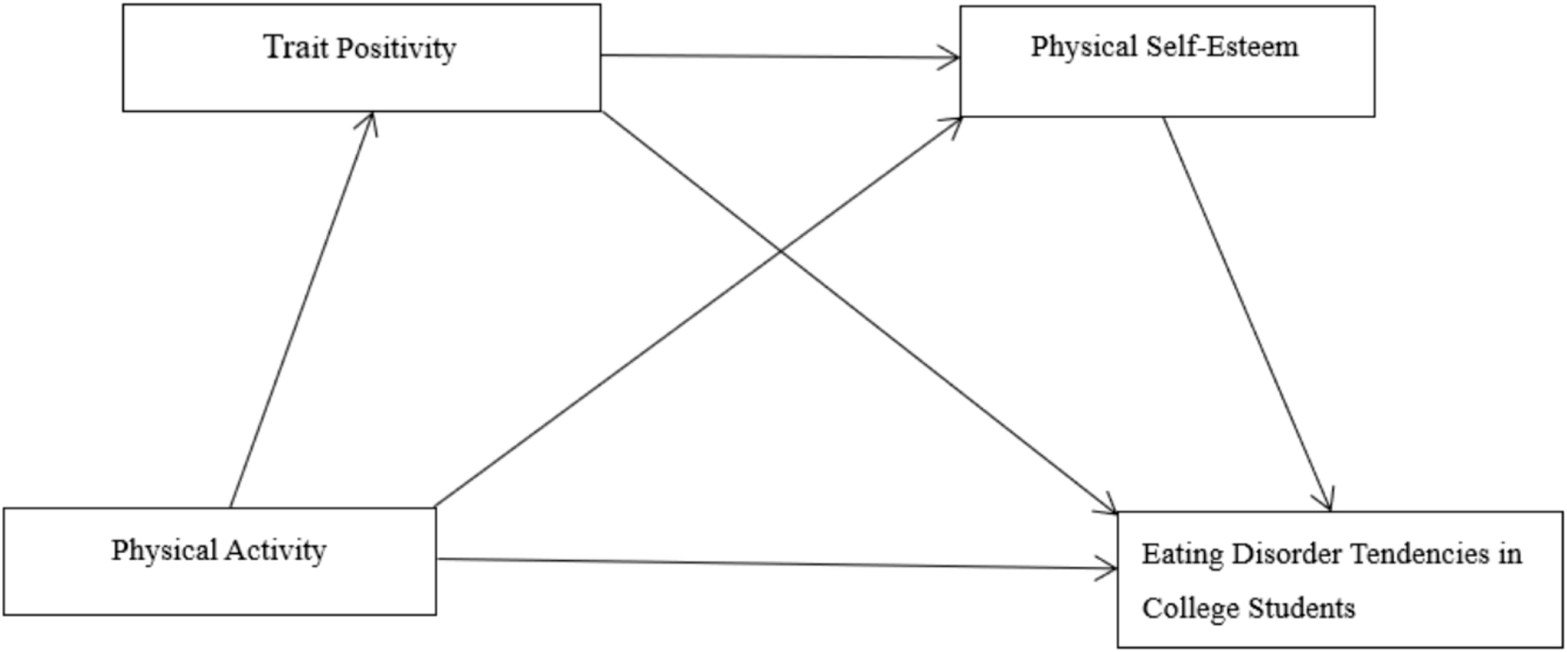
Diagram of the hypothetical model.

Trait positivity predicts physical self-esteem and regulates psychological well-being (Wang et al., 2020). As an ability to maintain awareness and focus on present-moment experiences, clinical psychology research confirms that trait positivity effectively improves negative thought patterns in patients with psychological disorders, thereby enhancing individuals’ physical self-esteem levels (Wang and Sun, 2018). Furthermore, health psychology studies indicate that trait positivity interventions mitigate the negative impact of psychological stress on physical self-esteem, serving as a protective factor for physical self-esteem (Yu, 2020). In educational psychology, trait positivity has been found to facilitate the development of positive self-perception in college students (Li and Zhang, 2019). Based on this, we propose Hypothesis H4: Trait positivity and physical self-esteem play a chain-mediated role between physical activity and eating disorder tendencies in college students (see Figure 1).

## 3 Methodology

### 3.1 Research subject

This study focused on exploring the effects of physical activity on eating disorder tendencies in college students and the chain mediating role of trait positivity and body esteem in. The study adopted a stratified random sampling strategy to select college students from different grades and distributed a total of 550 questionnaires to them. Among the recovered questionnaires, 17 questionnaires were excluded which were regarded as invalid due to extreme responses, incomplete responses or not completed within the specified time, and finally 533 valid questionnaires were successfully recovered, with a high validity recovery rate of 96.91%.

### 3.2 Research method

#### 3.2.1 Literature method

The literature method is the basis of this study. By systematically collecting and analysing literature in related fields, we aim to construct a theoretical framework and identify gaps and controversial points in the study. Various types of literature including academic journal articles, conference papers, official reports and books were widely collected to ensure a solid and comprehensive theoretical foundation for the study.

#### 3.2.2 Questionnaire survey method

Questionnaire survey method, as a quantitative research tool, collects data through a well-designed set of standardised questions. In this study, questionnaire survey method was used to collect data by selecting mature questionnaires and selecting participants through random sampling to ensure a representative sample, distributing the questionnaires and collecting them to collect the required data.

#### 3.2.3 Mathematical and statistical method

The study carried out data analysis with the help of SPSS 29.0 statistical software. At the preliminary stage of data processing, Cronbach’s alpha coefficient was applied to assess the reliability of the data and Harman’s one-way test was used to troubleshoot the possible problem of common method bias in the data collection process. The collected data were entered into SPSS software and the demographic characteristics of the sample were carefully analysed using descriptive statistics. In addition, the correlations between physical activity, eating disorder tendencies in college students, and trait positivity and body esteem were explored in depth by calculating Pearson correlation coefficients. Finally, a comprehensive analysis of the study was completed by performing multiple regression analyses using Model *6* in the PROCESS macro program and assessing the significance level of the mediating effect with the help of Bootstrap test.

### 3.3 Research instruments

#### 3.3.1 Physical activity level scale

The scale adopted in this study was developed by Japanese scholar Hashimoto Kimio (Liang, 1994) to assess participants’ physical activity during the previous week. This scale comprehensively evaluates three key dimensions of physical activity: intensity, duration, and frequency, using a 5-point Likert scale. According to the formula “Total score = Intensity × Frequency × Duration - 1,” higher scores indicate a higher level of physical activity (Han et al., 2024).

#### 3.3.2 Eating disorder tendency scale

This study employed the Eating Disorder Inventory (EDI) developed by Garner et al. (1983), a widely used screening tool for assessing individuals’ risk of eating disorder tendencies. The EDI-*3* Referral Form (EDI-3RF) version was adopted to identify individuals at high risk of eating disorder tendencies (Garner, 2004). This questionnaire comprises *25* items rated on a six-point scale, with higher total scores indicating more severe manifestations of eating disorder tendencies in the measured dimensions. The Cronbach’s alpha coefficient was 0.973.

#### 3.3.3 Philadelphia trait positivity scale

The Philadelphia Trait Positivity Scale was developed by Cardaciotto et al. (2008) based on Bishop et al.’s (2004) conceptualization, positing that trait positivity comprises two components: awareness and acceptance. Consequently, this scale conceptualizes trait positivity not as a second-order factor but as two distinct yet interrelated dimensions. Since this study emphasizes the acceptance-oriented aspect of trait positivity, this scale was selected to measure trait positivity. The Cronbach’s alpha coefficient was 0.751.

#### 3.3.4 Physical self-esteem scale

This study adopted the Body Esteem Scale (BES) revised by Chinese scholars Xu Xia and Yao Jiaxin for the psychological assessment of college students (Xu and Yao, 2001). The scale employs a 4-point rating system, with higher scores indicating a higher level of physical self-esteem. The Cronbach’s alpha coefficient was 0.951.

## 4 Research results

### 4.1 Control and test of common method bias

In this study, in order to ensure the accuracy of the data and to exclude the effect of common method bias, the Harman one-way test was used to test for common method bias. All scale questions were included in an exploratory factor analysis without rotation, and the test results showed that the number of factors with eigenvalues greater than 1 reached nine, indicating that the data were not dominated by a single factor, and that the variance explained by the largest factor was 26.124%, a percentage that is lower than the 40% critical criterion proposed by Podsakoff et al. (2003). Therefore, the data in this study were not affected by serious common method bias and the reliability of the results was guaranteed.

### 4.2 G-power test

To verify the support of the sample size for the chain-mediated model, a *post hoc* power analysis was performed using G-Power 3.1.9.2 with “F tests → Linear multiple regression.” The parameters were set as follows: effect size *f^2^ =* 0.504 (converted from the *R^2^* of the dependent variable), significance level *α =* 0.05, sample size *N =* 533, and number of predictors *k =* 3. The analysis showed that the power was 1.00 (see Table 1), indicating that the sample size was extremely sufficient to effectively detect the model effects.

**Table 1:** G-power analysis results.

G-power analysis parameters	Test family + statistical test	Type of power analysis	Effect size *f^2^*	*α*	Total sample size	Number of predictors	Power
Numerical Value	F Tests → linear multiple regression: fixed model, R^2^ deviation from zero	*Post hoc*: compute achieved power	0.504	0.05	533	3	1.00

Presents G-Power results for a linear multiple regression (fixed model, R^2^ deviation test). Key metrics: effect size f^2^ = 0.504 (large effect), *α* = 0.05, sample size *N* = 533, 3 predictors, and power = 1.00 (sufficient sample to detect effects).

### 4.3 Descriptive statistics

Demographic analysis showed that the sample size of this study was 533, with slightly more males than females, 52.5 and 47.5%, respectively. In terms of grade distribution, freshmen had the highest percentage of 57.6%, while graduate students had the lowest percentage of 7.1%. The proportions of sophomores, juniors and seniors were 15.0, 9.0 and 11.3%, respectively. This indicates that the sample is mainly concentrated on first-year students, with relatively small numbers of students in other grades (see Table 2).

**Table 2:** Demographic analysis.

Attribute	Category	Quantity	Percentage
Gender	Male	280	52.5%
	Female	253	47.5%
Grade	Freshman	307	57.6%
	Sophomore	80	15.0%
	Junior	48	9.0%
	Senior	60	11.3%
	Graduate students	38	7.1%

Breaks down sample (*N* = 533) by Gender (52.5% male, 47.5% female) and Grade (freshmen 57.6%, sophomores 15.0%, juniors 9.0%, seniors 11.3%, graduate students 7.1%). Shows demographic distribution.

When examining study variables, college students’ individual BMI values also correlate with other variables; therefore, data were analyzed separately by gender. First, participants’ age, height, and weight were grouped according to the categorization standards from the.

Chinese National Physical Fitness Monitoring Bulletin (General Administration of Sport of China, 2022) (results shown in Table 3). Subsequently, BMI values were calculated for each participant and grouped based on BMI magnitude according to Asian adult BMI classification criteria, with underweight (Group 1) defined as below 18.5, normal weight (Group 2) as 18.5 ~ 23.9, overweight (Group 3) as 24.0 ~ 27.9, moderate obesity (Group 4) as 28.0 ~ 32.4, and severe obesity (Group 5) as ≥32.5 (National Health Commission of the People’s Republic of China, 2024) (results shown in Table 4).

**Table 3:** Statistical analysis of height and weight data.

Gender	Age range	Quantity	Percentage	Height range (cm)	Quantity	Percentage	Weight range (kg)	Quantity	Percentage
Male (280)	<20	196	70.00%	<165	30	10.70%	<55	18	6.40%
	20–22	48	17.10%	165–180	180	64.30%	55–75	178	63.60%
	>22	36	12.90%	>180	70	25.00%	>75	84	30.00%
Female (253)	<20	173	68.40%	<155	10	4.00%	<50	47	18.60%
	20–22	44	17.40%	155–165	123	48.60%	50–65	148	58.50%
	>22	36	14.20%	>165	120	47.40%	>65	58	22.90%

Breaks down 533 participants by gender. Following national sports standards, males’ and females’ height/weight are each categorized into 3 levels. Shows quantity and percentage distributions.

**Table 4:** Statistical analysis of BMI data.

Attribute	Groups	Quantity	Percentage
Male	1	16	5.70%
	2	175	58.90%
	3	63	26.10%
	4	22	7.90%
	5	4	1.40%
Female	1	35	13.80%
	2	159	62.85%
	3	40	15.81%
	4	12	4.74%
	5	7	2.80%
Total		533	100%

BMI groups were defined as follows: Group 1 (Underweight): <18.5 kg/m^2^; Group 2 (Normal weight): 18.5–23.9 kg/m^2^; Group 3 (Overweight): 24.0–27.9 kg/m^2^; Group 4 (Moderate obesity): 28.0–32.4 kg/m^2^; Group 5 (Severe obesity): ≥32.5 kg/m^2^.

In addition, the sample was subjected to an independent samples t-test, which was first divided into two groups of men and women to compare whether there was a significant difference in each variable between the genders, and the results of the data measurements are shown in Table 5.

**Table 5:** Independent samples *t*-test.

Relevant Variable	Gender	*N*	*M ± SD*	*t*	*p*
Physical Activity	Male	280	20.293 ± 23.356	4.187	*p* < 0.001
	Female	253	12.210 ± 22.148		
Eating disorder tendencies	Male	280	3.404 ± 1.403	−4.395	*P* < 0.001
	Female	253	3.926 ± 1.334		
Trait positivity	Male	280	3.523 ± 0.541	3.181	*p* = 0.002
	Female	253	3.348 ± 0.720		
Physical self-esteem	Male	280	2.436 ± 0.436	7.154	*P* < 0.001
	Female	253	2.139 ± 0.522		

N denotes sample size, M ± SD represents mean ± standard deviation. T is the t-statistic and P is the *p*-value. A *p*-value < 0.05 indicates a statistically significant difference between groups.

The results of the independent samples *t*-test showed that there were significant differences between male and female groups across all four variables. Given that such gender differences may impact the relationship between physical activity and eating disorder tendencies, while also interfering with the chain - mediated effects of trait positivity and physical self - esteem, gender was included as a covariate in the subsequent regression and chain - mediated models. This was to control the potential influence of this confounding factor on the relationships among the core variables and ensure the accuracy and validity of the research findings.

### 4.4 ANOVA single factor analysis

Firstly, ANOVA one-way analysis was used to analyse the differences in physical activity, eating disorder tendency, trait positivity and Physical Self-Esteem among college students of different grades. The results showed that freshmen and junior and senior students showed significant differences in the physical activity dimension. As shown in Table 6.

**Table 6:** One-way analysis of ANOVE for college students of different grades.

Relevant variable	Grade	*M ± SD*	*F*	*P*
Physical activity	1 (307)	11.769 ± 15.465	18.250	P<0.001
	2 (80)	14.038 ± 18.558		
	3 (48)	18.542 ± 23.804		
	4 (60)	28.850 ± 34.194		
	5 (38)	34.211 ± 33.980		
Eating disorder tendencies	1 (307)	3.452 ± 1.351	7.378	*P<*0.001
	2 (80)	3.649 ± 1.330		
	3 (48)	4.483 ± 1.282		
	4 (60)	4.044 ± 1.437		
	5 (38)	3.560 ± 1.489		
Trait positivity	1 (307)	3.392 ± 0.619	3.301	0.011
	2 (80)	3.416 ± 0.666		
	3 (48)	3.430 ± 0.712		
	4 (60)	3.714 ± 0.561		
	5 (38)	3.453 ± 0.664		
Physical self-esteem	1 (307)	2.255 ± 0.462	8.200	*P <* 0.001
	2 (80)	2.119 ± 0.486		
	3 (48)	2.468 ± 0.522		
	4 (60)	2.506 ± 0.494		
	5 (38)	2.435 ± 0.628		

M ± SD indicates mean ± standard deviation. F is the F-statistic for testing the null hypothesis of equal group means. P is the *p*-value; *p* < 0.05 suggests statistically significant differences among groups.

To clearly present differences in physical activity, eating disorder tendencies in college students, trait positivity, and physical self-esteem across academic years, multiple comparison analyses were conducted with the following results:

Physical Activity: Significant differences emerged between graduate students and freshmen (mean difference = −25.44180*), sophomores (−23.17303*), and juniors (−18.66886*); between seniors and freshmen (−17.08127*), sophomores (−14.81250*), and juniors (−10.30833*). No significant differences were observed between juniors and freshmen/sophomores, or between sophomores and freshmen (* Indicates significant mean difference at the 0.05 level. Means for each grade are shown in parentheses, and the same applies hereinafter).
Eating Disorder Tendencies in College Students: Significant differences existed between graduate students and juniors (−0.88439*); seniors and freshmen (−0.59175*); juniors and freshmen (−1.03109*) or sophomores (−0.83483*). All other inter-year comparisons were non-significant.
Trait Positivity: Significant differences occurred between seniors and freshmen (−0.32209*), and juniors and sophomores (−0.29792*). All other comparisons were non-significant.
Physical Self-Esteem: Significant differences were found between postgraduate students and freshmen (−0.18026*) or sophomores (−0.31592*); seniors and freshmen (−0.25072*) or sophomores (−0.38639*); juniors and freshmen (−0.21322*) or sophomores (−0.34889*); and sophomores and freshmen (0.13567). All other inter-year differences were non-significant.

Secondly, men and women were divided into two groups according to gender, and each group was analysed by ANOVA one-way analysis of the differences between college students with different BMI levels in each variable. The data results of male college students in different BMI level groups are shown in Table 7.

**Table 7:** ANOVA one-way analysis of male college students grouped by BMI value.

Relevant variable	Groups	*M ± SD*	*F*	*P*
Physical activity	1 (16)	13.375 ± 12.317	0.657	*p =* 0.622
	2 (175)	20.152 ± 23.143		
	3 (63)	22.795 ± 23.529		
	4 (22)	18.591 ± 18.076		
	5 (4)	17.500 ± 20.744		
Eating disorder tendencies	1 (16)	3.068 ± 1.544	0.748	*p =* 0.560
	2 (175)	3.490 ± 1.463		
	3 (63)	3.335 ± 1.355		
	4 (22)	3.375 ± 1.038		
	5 (4)	2.610 ± 0.794		
Trait positivity	1 (16)	3.350 ± 0.596	0.560	*p =* 0.692
	2 (175)	3.549 ± 0.508		
	3 (63)	3.509 ± 0.627		
	4 (22)	3.511 ± 0.483		
	5 (4)	3.413 ± 0.287		
Physical self-esteem	1 (16)	2.392 ± 0.458	1.282	*p =* 0.277
	2 (175)	2.442 ± 0.438		
	3 (63)	2.474 ± 0.406		
	4 (22)	2.259 ± 0.485		
	5 (4)	2.625 ± 0.513		

M ± SD indicates mean ± standard deviation. F is the F-statistic for testing the null hypothesis of equal group means. P is the *p*-value; *p* < 0.05 suggests statistically significant differences among groups.

One - way ANOVA results indicated that, for the male college student population, there were no statistically significant differences in the inter - group comparisons of the four variables s, namely Physical Activity, Eating Disorder Tendencies, Trait Positivity, and Physical Self - Esteem, among different BMI groups. Since the overall inter - group differences in the one - way ANOVA did not reach the level of statistical significance (*p* > 0.05), there was no need to conduct post - hoc tests to further explore the specific differences between groups.

The results of the data of female college students in different BMI level groups are shown in Table 8.

**Table 8:** ANOVA one-way analysis of female college students grouped by BMI value.

Relevant variable	Groups	*M ± SD*	*F*	*P*
Physical activity	1 (35)	10.543 ± 21.597	0.675	*p =* 0.610
	2 (159)	13.541 ± 24.387		
	3 (40)	9.350 ± 14.414		
	4 (12)	14.250 ± 19.564		
	5 (7)	3.143 ± 4.880		
Eating disorder tendencies	1 (35)	3.984 ± 1.547	0.830	*p =* 0.507
	2 (159)	3.964 ± 1.291		
	3 (40)	3.884 ± 1.283		
	4 (12)	3.270 ± 1.175		
	5 (7)	4.149 ± 1.763		
Trait positivity	1 (35)	3.290 ± 0.775	0.721	*p =* 0.578
	2 (159)	3.376 ± 0.707		
	3 (40)	3.210 ± 0.730		
	4 (12)	3.492 ± 0.714		
	5 (7)	3.536 ± 0.741		
Physical self-esteem	1 (35)	1.975 ± 0.535	1.319	*p =* 0.263
	2 (159)	2.175 ± 0.525		
	3 (40)	2.131 ± 0.505		
	4 (12)	2.242 ± 0.394		
	5 (7)	1.991 ± 0.626		

M ± SD indicates mean ± standard deviation. F is the F-statistic for testing the null hypothesis of equal group means. P is the *p*-value; *P* < 0.05 suggests statistically significant differences among groups.

One - way ANOVA results indicated that, for the female college student population, there were no statistically significant differences in the inter - group comparisons of the four variables, namely Physical Activity, Eating Disorder Tendencies, Trait Positivity, and Physical Self - Esteem, among different BMI groups. Given that the overall inter - group differences in the one - way ANOVA did not reach statistical significance (*p* > 0.05), post - hoc tests were not conducted to further examine the specific differences between groups.

### 4.5 Correlation analysis between variables

Using SPSS software, correlation analyses were performed by importing the four variables—physical activity, eating disorder tendencies in college students, trait positivity, and physical self-esteem—into the dataset. Calculation of Pearson correlation coefficients (Table 9) revealed a significant negative correlation between physical activity and eating disorder tendencies in college students, indicating that higher frequency of participation in physical activity corresponds to a lower likelihood of developing eating disorder tendencies. Furthermore, physical activity showed significant positive correlations with both trait positivity and physical self-esteem, suggesting that individuals who regularly engage in physical activity tend to exhibit higher levels of trait positivity and physical self-esteem. Simultaneously, both trait positivity and physical self-esteem demonstrated significant negative correlations with eating disorder tendencies in college students, implying that higher levels of trait positivity and stronger physical self-esteem are associated with lower eating disorder tendencies. The results indicate that physical activity, trait positivity, and physical self-esteem play important roles in preventing and intervening in eating disorder tendencies in college students.

**Table 9:** Person correlation analysis.

Relevant Variable	Physical activity	Eating disorder tendencies	Trait positivity	Physical self-esteem
Physical activity	1			
Eating disorder tendencies	−0.361**	1		
Trait positivity	0.223**	−0.275**	1	
Physical self-esteem	0.371**	−0.436**	0.433**	1

Significant correlation at the 0.01 level (two-tailed).

To investigate the relationships among physical activity (*X*), demographic variables (gender, academic year, BMI groups), trait positivity (*M1*), physical self-esteem (*M2*), and eating disorder tendencies in college students (*Y*), this study employed hierarchical multiple linear regression analysis with stepwise entry of predictors. Using trait positivity, physical self-esteem, and eating disorder tendencies in college students as dependent variables, we sequentially examined the explanatory power of physical activity and demographic variables. Model fit indices and significance levels of regression coefficients are presented in Table 10. Results demonstrated that physical activity and specific demographic variables significantly predicted trait positivity and physical self-esteem, with a chain-mediated effect through trait positivity → physical self-esteem ultimately influencing eating disorder tendencies in college students. These findings provide empirical evidence for elucidating the psychological mechanisms underlying eating behaviors in college students.

**Table 10:** Regression analysis between variables.

Regression equation		Overall fit index			Significance of regression coefficients		
Outcome variable	Predictor variable	*R*	*R^2^*	*F*	*β*	*t*	*p*
Trait positivity (*M1*)	Physical activity (*X*)	0.251 0.063 8.864			0.188	4.110	<0.001
	Gender				−0.101	−2.308	0.021
	Grade				0.050	1.108	0.268
	BMI group				0.036	0.840	0.401
Physical self-esteem (*M2*)	Physical activity (*X*)	0.557 0.311 47.483			0.233	5.830	<0.001
	Trait positivity *(M1)*				0.345	9.242	<0.001
	Gender				−0.212	−5.644	<0.001
	Grade				0.069	1.779	0.076
	BMI Group				−0.008	−0.221	0.825
Eating disorder tendencies *(Y)*	Physical activity *(X)*	0.579 0.335 44.233			−0.328	−8.091	<0.001
	Trait positivity *(M1)*				−0.093	−2.343	0.020
	Physical self-esteem *(M2)*				−0.330	−7.706	<0.001
	Gender				−0.001	−0.012	0.990
	Grade				0.323	8.455	<0.001
	BMI Group				−0.024	−0.659	0.510

“Overall Fit Index” includes R (correlation), R^2^ (variance explained), F (model significance test). “Significance of Regression Coefficients” has β (effect size), t (individual predictor test), P (*P* < 0.05 indicates significant association).

### 4.6 Chain-mediated effects of trait positivity and physical self-esteem between physical activity and eating disorder tendencies

**Figure 2:**
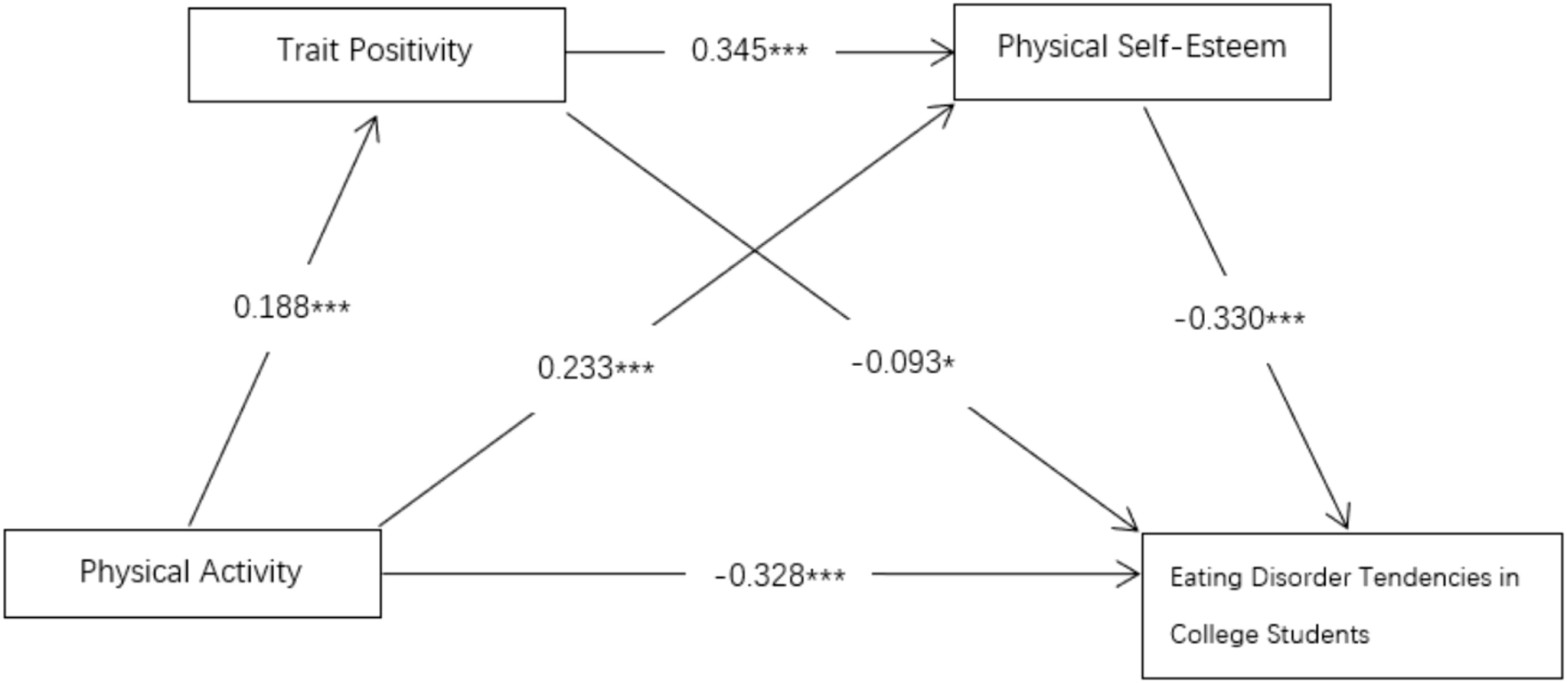
Chain brokerage model diagram. The values in this figure were generated from the *β* and *p* in Table 10. In this figure, *p*-values less than 0.001 are represented by three stars, indicating high significance; *p*-values less than 0.01 are shown as two stars, denoting extreme significance; and *p*-values less than 0.05 are marked by one star, indicating significance.

Trait positivity and physical self-esteem act as chain mediators between physical activity and eating disorder tendencies. Physical activity reduces eating disorder tendencies by enhancing trait positivity, which in turn enhances physical self-esteem. This chain-mediated effect suggests that physical activity not only directly positively affects eating disorder tendency, but also indirectly affects eating disorder tendency through the psychological machines (trait positivity and physical self-esteem), the data results of which are shown in Table 11, and the model diagram is shown in Figure 2.

**Table 11:** Chain mediation effects table.

Effect (scientific phenomenon)	Trails	Efficiency value	Standard error	*LLCL*	*ULCL*	Efficiency ratio
Aggregate effect		−0.0273	0.0026	−0.0324	−0.0222	100%
Direct effect	Direct Path	−0.0202	0.0025	−0.0251	−0.0153	73.99%
Total indirect effect		−0.0071	0.0013	−0.0098	−0.0049	26.01%
Indirect effect	Path 1	−0.0011	0.0005	−0.0022	−0.0002	4.02%
	Path 2	−0.0047	0.0011	−0.0070	−0.0028	17.23%
	Path 3	−0.0013	0.0004	−0.0022	−0.0006	4.76%

Table 11 shows chain mediation effects. Columns: effect types, paths, effect size, variability, significance (via LLCL/ULCL), and ratio of total effect.

## 5 Discussion

### 5.1 Group difference analyses

Using independent samples t-tests, results indicated that males generally engaged in physical activity more actively, while females exhibited higher vulnerability to eating disorder tendencies in college students, consistent with prior research (Beaulieu and Best, 2024). Variations in hormone levels may contribute to gender differences in eating disorder tendencies among college students (Versini et al., 2010). Additionally, females’ emphasis on perfectionism (Culbert et al., 2021) and self-objectification psychology (Fredrickson and Roberts, 1997) were key factors. Regarding trait positivity, males demonstrated greater present-moment focus and reduced reactivity to negative emotions, potentially linked to hormonal differences, gender-specific emotion regulation patterns, and females’ higher self-compassion levels (Bleidorn et al., 2016). For physical self-esteem, males reported more positive body image evaluations and fewer body image concerns (Gualdi-Russo et al., 2022).

Univariate ANOVA across academic years revealed significantly increased physical activity scores in higher grades, particularly from junior to postgraduate stages. This may reflect enhanced time management skills, greater health awareness, and proactive health behaviors (Carducci et al., 2020), alongside increased leisure time and resource access (Guo et al., 2024). Eating disorder tendencies peaked during junior year, potentially due to academic pressure and career uncertainty prompting maladaptive eating behaviors (Yang, 2011), then decreased in senior and postgraduate years as future plans solidified (Zheng, 2024). Trait positivity scores were highest among seniors, possibly indicating stress-coping strategies during graduation transitions (Ouyang et al., 2019), while stable scores in lower grades suggested underdeveloped mindfulness habits (Hu and Gu, 2024). Physical self-esteem improved in junior/senior years, reflecting increased body acceptance, whereas freshmen/sophomores showed lower scores due to heightened appearance focus and social evaluation (Zhang and Song, 2008).

In this study, no significant differences were observed across various variables among different BMI groups within male and female college student populations. This may be attributed to the interactive effects of multiple factors. At the physiological level, as a broad body shape indicator, BMI fails to fully capture the subtle differences in body composition (such as muscle mass and body fat distribution) within genders among college students, limiting its predictive power for behavioral and psychological variables (Verma et al., 2016). In terms of environment, uniform campus dietary and exercise resources (Zhou and Zeng, 2025) may weaken the potential impact of BMI on physical activity and eating behavior. Additionally, external factors such as academic stress and social culture may have a stronger driving effect on psychological traits (such as trait mindfulness and physical self-esteem) in both genders (Woolley, 1903). Regarding psychological traits, gender-specific psychological tendencies, such as females’ focus on body image (Zhao, 2022) and males’ pursuit of achievement (Yang Q. W. et al., 2023), may obscure the role of BMI. It is worth noting that this finding diverges from previous studies on adult populations (Li, 2024; Zhang, 2024), which may stem from the unique developmental stage characteristics of college students, altering the influence pathway of BMI on behavior and psychology in this group. However, this study is limited to college students and uses BMI as a single grouping indicator, failing to comprehensively consider key body composition factors such as body fat percentage and muscle mass, which may affect the interpretation of results. Therefore, this study has limitations. Future research could incorporate more refined body measurement indicators, adopt longitudinal tracking designs, and expand sample diversity to deeply explore the relationships among BMI, health behaviors, and psychological variables.

### 5.2 Main effect analysis of physical activity on eating disorder tendencies in college students

Results demonstrated a significant negative correlation between physical activity and eating disorder tendencies in college students, with physical activity effectively and negatively predicting eating disorder tendencies, thereby validating Hypothesis H1. Specifically, physical activity actively contributes to the treatment of binge eating disorder, significantly reducing binge eating behaviors and associated symptoms (Guerriero et al., 2025). Furthermore, physical activity aids in effectively managing compulsive exercise behavior—a key risk factor for eating disorder tendencies (Li et al., 2024).

As an efficacious psychological intervention, physical activity significantly enhances emotion regulation. It effectively alleviates anxiety and stress, which frequently trigger eating disorder tendencies (Zhao et al., 2024). Social pressure also constitutes a critical risk factor for eating disorder tendencies (Monteleone et al., 2020), physical activity mitigates this pressure by improving individuals’ adaptability to social evaluation, thereby reducing eating disorder tendencies induced by social stressors (Wu et al., 2025). Additionally, physical activity provides a positive social environment conducive to establishing healthier interpersonal relationships (Zhang, 2023).

Universities should actively encourage student participation in physical activity and offer diversified physical activity programs alongside trait positivity training courses to comprehensively promote students’ physical and mental health. This approach not only prevents and reduces eating disorder tendencies in college students but also enhances their overall quality of life.

### 5.3 Chain-mediated effect analysis of trait positivity and physical self-esteem

This study constructed a model with trait positivity and physical self-esteem as mediators to examine how physical activity influences eating disorder tendencies in college students. Results confirmed that trait positivity mediated the relationship between physical activity and eating disorder tendencies in college students, validating Hypothesis H2. Physical activity significantly and positively predicted trait positivity levels. Research indicates that physical activity enhances psychological well-being by elevating trait positivity (Sala et al., 2022). Individuals with higher trait positivity exhibit protective effects against pathological mechanisms of eating disorder tendencies, characterized by improved emotional expression, acceptance of discomfort during eating, and reduced hyperfocus on food/body sensations—processes significantly linked to lower eating disorder tendencies (Giannopoulou et al., 2020). Concurrently, trait positivity training effectively manages compulsive exercise behavior (Bacalhau et al., 2025), a critical risk factor for eating disorder tendencies (Lichtenstein et al., 2017).

Second, findings revealed the mediating role of physical self-esteem between physical activity and eating disorder tendencies, confirming Hypothesis H3. Physical activity significantly and positively predicted physical self-esteem. Physically active individuals held more positive body perceptions, enhancing physical self-esteem (Yang C. et al., 2023), while higher physical self-esteem showed a significant negative correlation with eating disorder tendencies. Satisfactory body image substantially reduced eating disorder tendencies risk (Gao et al., 2024), improving mental health and overall quality of life. Enhanced physical self-esteem further strengthened school adaptability and psychological resilience (Winn and Cornelius, 2020), underscoring the imperative for educational policymakers to integrate physical activity into curricula.

Finally, the chain-mediated effect of trait positivity and physical self-esteem between physical activity and eating disorder tendencies in college students was confirmed (Hypothesis H4). Beyond direct effects, physical activity reduced eating disorder tendencies by elevating trait positivity (Zhao and Yin, 2024), thereby boosting physical self-esteem. Physiologically, trait positivity reduces stress-induced abnormal activation of body perception-related neural circuits, promoting objective awareness of the body (Yu et al., 2012); psychologically, trait positivity fosters acceptance of body image, shifting focus from flaws to positive physical experiences, thereby strengthening physical self-esteem (Xu, 2022). Individuals with higher trait positivity demonstrated greater physical self-esteem due to reduced stress reactivity, enhanced emotional stability, and increased body acceptance (Grepmair et al., 2007), mitigating body dissatisfaction. Higher physical self-esteem subsequently lowered vulnerability to eating disorder tendencies (Tian and Yang, 2024). Thus, physical activity indirectly reduced eating disorder tendencies through three pathways: the specific mediating role of trait positivity, the specific mediating role of physical self-esteem, and their chain-mediated effect. This chain-mediated mechanism provides novel insights into the physical activity–eating disorder tendencies relationship and offers empirical support for campus-based mental health interventions.

### 5.4 Practical implications and recommendations

Society should advocate for diverse aesthetic standards, avoiding the promotion of a singular “thin-ideal” beauty norm (Schroeder and Behm-Morawitz, 2024). Media must highlight healthy lifestyles across diverse body types and genders to foster inclusivity (Du and He, 2025). Communities should organize varied physical activity programs to encourage equal participation and dismantle gender stereotypes. Public awareness campaigns should increase understanding of eating disorder tendencies and related psychological issues, ensuring accessible professional counseling services.

In terms of educational institutions, they should fully leverage their guiding and educational roles. Based on the differences among college students of different grades in physical exercise, eating disorder tendencies, trait positivity, and physical self-esteem, the recommendations are as follows: strengthen the promotion of sports clubs and organize activities for lower-grade students (freshmen and sophomores) to cultivate exercise habits; incorporate a “Body Image and Healthy Diet” module integrated with trait positivity practices into the mental health courses for sophomores and juniors; implement grade-specific trait positivity curricula, establish workshops for sophomores, and engage senior students as “trait positivity peers”; combine trait positivity training with physical self-esteem cultivation, design specialized courses for lower-grade students, and integrate trait positivity elements into physical education teaching to promote physical and mental well-being.

College students ought to boost self - awareness and cultivate proper aesthetic and value systems to mitigate negative sociocultural impacts (Pan, 2025). Since physical activity can build a healthy lifestyle and augment physical self - esteem and psychological resilience, they should engage in sports actively. Managing emotions and alleviating stress via methods like Trait Positivity training and meditation is beneficial for psychological well - being (Zhang, 2025). Students need to focus on their own requirements and promptly seek professional assistance to avert the worsening of psychological issues.

## 6 Conclusion

Physical activity had a significant indirect effect on eating disorder tendencies in college students by enhancing trait positivity and physical self-esteem. This chain-mediated effect not only provides a new perspective for understanding the relationship between physical activity and eating disorder tendencies, but also provides empirical support for colleges and universities in promoting students’ mental health and preventing eating disorder tendencies. By encouraging students to participate in physical activity and combining it with trait positivity training, students’ trait positivity and physical self-esteem levels can be effectively enhanced, which in turn reduces eating disorder tendencies and promotes students’ overall health and quality of life.

## Data Availability

The raw data supporting the conclusions of this article will be made available by the authors, without undue reservation.
